# *Achromobacter* Species Isolated from Cystic Fibrosis Patients Reveal Distinctly Different Biofilm Morphotypes

**DOI:** 10.3390/microorganisms4030033

**Published:** 2016-09-14

**Authors:** Signe M. Nielsen, Niels Nørskov-Lauritsen, Thomas Bjarnsholt, Rikke L. Meyer

**Affiliations:** 1 Department of Clinical Medicine, Aarhus University, DK-8200 Aarhus, Denmark; smni@clin.au.dk; 2 Department of Clinical Microbiology, Aarhus University Hospital, DK-8200 Aarhus, Denmark; nielnoer@rm.dk; 3 Costerton Biofilm Center, Department of Immunology and Microbiology, University of Copenhagen, DK-2200 Copenhagen, Denmark; tbjarnsholt@sund.ku.dk; 4 Department of Clinical Microbiology, Rigshospitalet, DK-2100 Copenhagen, Denmark; 5 Interdisciplinary Nanoscience Center, Aarhus University, DK-8000 Aarhus, Denmark; 6 Department of Bioscience, Aarhus University, DK-8000 Aarhus, Denmark

**Keywords:** *Achromobacter*, cystic fibrosis, biofilm, biofilm morphology, dispersal, confocal microscopy, PNA-FISH, antimicrobial susceptibility testing, J0101

## Abstract

*Achromobacter* species have attracted attention as emerging pathogens in cystic fibrosis. The clinical significance of *Achromobacter* infection is not yet fully elucidated; however, their intrinsic resistance to antimicrobials and ability to form biofilms renders them capable of establishing long-term chronic infections. Still, many aspects of *Achromobacter* biofilm formation remain uncharacterized. In this study, we characterized biofilm formation in clinical isolates of *Achromobacter* and investigated the effect of challenging the biofilm with antimicrobials and/or enzymes targeting the extracellular matrix. In vitro biofilm growth and subsequent visualization by confocal microscopy revealed distinctly different biofilm morphotypes: a surface-attached biofilm morphotype of small aggregates and an unattached biofilm morphotype of large suspended aggregates. Aggregates consistent with our in vitro findings were visualized in sputum samples from cystic fibrosis patients using an *Achromobacter* specific peptide nucleic acid fluorescence in situ hybridization (PNA-FISH) probe, confirming the presence of *Achromobacter* biofilms in the CF lung. High antibiotic tolerance was associated with the biofilm phenotype, and biocidal antibiotic concentrations were up to 1000 fold higher than for planktonic cultures. Treatment with DNase or subtilisin partially dispersed the biofilm and reduced the tolerance to specific antimicrobials, paving the way for further research into using dispersal mechanisms to improve treatment strategies.

## 1. Introduction

Cystic fibrosis (CF) is an inherited chronic disease characterized by the development of chronic pulmonary infections caused by increased viscosity of bronchial mucus and impaired mucocilliary clearance of inhaled bacteria [1,2,3]. *Achromobacter* sp. are increasingly isolated from cystic fibrosis patients. Although the increased prevalence has partially been ascribed to improved identification techniques and increased attention to the identification of *Achromobacter* species, other important factors such as aggressive antimicrobial treatment and prolonged lifespan of the patients may also have given rise to increased prevalence rates. As well as being innately resistant to many antimicrobial agents, *Achromobacter* may also develop resistance to virtually all available antibiotics [4,5,6,7], and have attracted attention as emerging pathogens in cystic fibrosis [8]. Several species within the genus *Achromobacter* have been isolated from cystic fibrosis patients; the most common being *Achromobacter* *xylosoxidans* however, other *Achromobacter* species may also cause infections in these patients [9,10].

*A. xylosoxidans* has the capacity to form biofilm [11,12], and biofilm formation is a key aspect of the chronic lung infections of cystic fibrosis patients. Biofilms are diverse structures with variable morphology, growing either attached to a surface or as unattached aggregates [13,14]. In a biofilm, bacterial cells aggregate into micro colonies embedded in a matrix of extracellular polymeric substances (EPS). The components and the amount of EPS often vary depending on the bacterial species, the environmental conditions, and the maturation stage of the biofilm [15].

The matrix provides a protective environment, shielding bacteria against antimicrobials and host defense systems [16,17]. Furthermore, bacteria commonly reduce their metabolic activity when in a biofilm, a trait further enhancing their antimicrobial tolerance [18]. Taken together, these factors lead to difficulties when treating cystic fibrosis patients, rendering biofilm infections in the lungs of these patients hard if not impossible to eradicate, thus leading to chronic pulmonary infections; the main cause of morbidity and mortality in cystic fibrosis patients. The clinical impact of *Achromobacter* biofilm formation is still unclear, and the literature regarding *Achromobacter* largely consists of case reports while only few studies regarding *Achromobacter* in vitro biofilm formation and antimicrobial susceptibility have been published [11,19]. Many aspects of *Achromobacter* biofilm formation thus remain to be investigated. Eradication of biofilm infections often fail despite aggressive antimicrobial treatment. A better understanding of the capacity of *Achromobacter* to form biofilm may help improve diagnosis and treatment strategies of *Achromobacter* in cystic fibrosis patients. In this study, we investigated biofilm formation in clinical isolates of *Achromobacter* obtained from chronically infected cystic fibrosis patients. The aim was to gain further insight into fundamental aspects of the biofilm morphology and composition of clinical *Achromobacter* isolates, and to investigate the effect of challenging the biofilm with antimicrobials and/or enzymes targeting the extracellular matrix.

## 2. Materials and Methods

### 2.1. Bacterial Strains

Isolates of the clinical *Achromobacter* strains used in this study were obtained from airway secretions collected from cystic fibrosis patients during routine investigation at the CF center at Aarhus University Hospital, Denmark. Identification to species level of the strains used in this study had previously been performed using Multilocus Sequence analysis (MLSA) [20,21]. Biofilm formation was investigated for 23 *Achromobacter* isolates and one isolate of *P. aeruginosa* PAO1 DSMZ 19880 (Table 1). Three *Achromobacter* isolates were obtained from culture collections; two type strains (*A. xylosoxidans* LMG 1863 and *A. ruhlandii* CCUG 38886), and one environmental isolate (*A. xylosoxidans* CCUG 14603). Fifteen of the 20 clinical *Achromobacter* isolates originated from five CF patients [21], with three isolates from each: One isolate “a” was a first time isolate obtained the first time an *Achromobacter* was cultured from a routine sputum sample. Isolate “b” was selected approximately one year after the patient was first infected, and the third isolate “c” was selected approximately four years after the patient was first infected with *Achromobacter*. The “b” and “c” isolates are henceforth referred to as “later” isolates. The isolates were identified as *A. ruhlandii* (CF1), *A. xylosoxidans* (CF2) and *A.* *insuavis* (CF3-CF5). Five additional isolates originated from five different patients; three isolates of *A. xylosoxidans* (CF6-CF8) and two isolates of *A. ruhlandii* (CF9 and CF10). Pulsed-field gel electrophoresis typing of isolates showed that CF9 and CF10 both belonged to the same clone, the Danish Epidemic Strain (DES) of *A. ruhlandii* [22,23], and thus represented putative cases of patient-to-patient transmission. All other isolates were unique (S. Gade, N. Nørskov-Lauritsen and W. Ridderberg, submitted for publication). Isolates were cultured on 5% blood agar at 37 °C. Planktonic cultures were obtained by inoculating sterile media (Brain Heart Infusion (BHI) broth (Fluka) or ABT minimal medium containing 0.5% glucose) with a single colony and incubating at 37 °C overnight in Erlenmeyer flasks with shaking at 180 rpm. Prior to each experiment, overnight cultures were adjusted to OD600 of 0.1 using fresh growth media, corresponding to approximately 106 CFU/mL.

### 2.2. Fast Screening of Biofilm Formation

All strains were subjected to quantification of biofilm formation using a crystal violet micro titer assay, modified and adapted for adherence to peg-lid (Nunc-immuno™ TSP) [24,25]. In brief, overnight cultures were adjusted to OD600 = 0.1 with fresh BHI, and 160 µL per well was added to 96 well micro titer plates (Nunc, Nunclon Delta Surface, Thermo Fischer Scientific, Waltham, MA, USA). Peg-lids were inserted and plates were incubated statically for 24 h at 37 °C. Following incubation, the peg-lids were gently immersed in water to remove unbound bacteria and inserted into 96 well plates containing 180 µL 1% crystal violet solution in water and incubated for 15 min to stain the biofilm adhering to the pegs. The pegs were then washed three times by gently immersing them in water to wash off unbound crystal violet and left to air-dry for 20 min. Crystal violet was extracted by submerging the pegs in 96% ethanol (180 µL in each well) for 15 min, and the amount of biofilm was determined by optical density measurements of crystal violet at 585 nm using a BioTek Power wave XS2 plate reader (Holm & Halby, Brøndby, Danmark). The experiment was carried out with three biological and eight technical replicates. The result of this screening procedure was used to select strains for further characterization.

Growth rates of individual strains were determined by optical density measurements at 600 nm during growth of planktonic cultures in BHI in 96 well plates with 200 µL in each well. The bacteria were grown at 37 °C with shaking. Measurements were taken every 30 min using a Multiskan™ GO Microplate Spectrophotometer (Thermo Fisher Scientific) in kinetic mode.

### 2.3. Characterisation of Biofilms Grown under Continuous Flow

For characterization of structure and matrix composition, biofilms of *A. xylosoxidans* LMG 1863^T^, *A. xylosoxidans* CF2-b and *A. insuavis* CF4-b were grown under continuous flow as described [26]. Briefly, planktonic cultures of each strain were grown overnight in ABT media. One mL was injected into a flow chamber (µ-Slide VI 0.4, ibidi GmbH, Martinsried, Germany) connected to a peristaltic pump. The cells were left to attach for 1½ h without flow. Biofilms were cultured for 48 h at 37 °C during continuous flow in ABT media with a flow rate of 1.5 mL/h. The biofilms were stained by injecting stains into the flow chamber. LIVE/DEAD^®^ stain (BacLight L7007 bacterial viability kit for microscopy, Invitrogen, Thermo Fisher, Waltham, MA USA) was prepared according to the manufacturer’s protocol, with the modification that the concentration of propidium iodide was increased to 0.05 mM, in order to stain extracellular DNA [27]. Three µL LIVE/DEAD^®^ stain diluted in 1 mL 0.85% NaCl was injected into the flow chamber. The biofilm was stained for 15 min. Thereafter, the biofilm was stained with Calcofluor (100 mg/mL in 0.85% NaCl) (Sigma-Aldrich, St. Louis MO, USA) for fifteen min and rinsed with 0.85% NaCl for fifteen min at continuous flow prior to imaging using confocal laser scanning microscopy (CLSM) (Zeiss LSM 700, Carl Zeiss AG, Oberkochen, Germany).

### 2.4. Characterisation of Statically Grown Biofilms

*A. insuavis* CF4-b did not produce an attached biofilm and could therefore not be studied under flow. To characterize the structure and matrix composition of unattached biofilm, *A. insuavis* CF4-b was grown in planktonic culture overnight in ABT media. A microscopy dish (µ-Dish 35 mm, high, ibidi GmbH, Martinsried, Germany) was inoculated with two mL planktonic and incubated for 48 h at 37 °C. Following incubation, growth media was carefully removed paying close attention to not disturbing the aggregated structures. The microscopy dish was gently rinsed twice with two mL PBS to remove planktonic bacteria. Biofilm aggregates were stained for 15 min by carefully adding one mL LIVE/DEAD^®^ stain as described above. The biofilm was then stained with Calcofluor for 15 min, and rinsed three times with one mL 0.85% NaCl. Finally, two mL of an 80% glycerol solution was added to stabilize the biofilm prior to imaging using confocal microscopy.

### 2.5. Detection of Achromobacter Aggregates in Sputum Samples from Cystic Fibrosis Patients Using Peptide Nucleic Acid (PNA) Fluorescent in Situ Hybridization (FISH)

PNA-FISH was performed on sputum samples to evaluate the presence and mode of growth of *Achromobacter* in order to compare with in vitro biofilm formation. The sputum samples were obtained from the two CF patients colonized with *A. xylosoxidans* CF2 and *A. insuavis* CF4, following routine examination. *Achromobacter* specific FISH probe Ach-221 [28] was modified to PNA-FISH (5’-CGC TCY AAT AGT GCA AGG TC-3’ labeled with Cy3), by Eurogentec (EGT North America, oligonucleotides, Campus Drive, Freemont, CA, USA).

### 2.6. Probe Validation on Pure Cultures

The specificity of the *Achromobacter* PNA-FISH probe was validated against a negative control strain with two nucleotide mismatches (*Neisseria polysaccharea* DSM 22809), and *P. aeruginosa* PAO1 with *A. xylosoxidans* CF2-b serving as positive control (Table 1) using seven different hybridization buffers with increasing formamide concentration (0%, 5% 10% 15% 20% 25% or 30% (vol/vol) formamide). Hybridization buffer contained 10% (wt/vol) dextran sulphate, 10 mM NaCl, 0.1% (wt/vol) sodium pyrophosphate 0.2% (wt/vol) polyvinylpyrrolidone, 0.2% (wt/vol) ficoll, 5 mM disodium EDTA, 0.1% (vol/vol) Triton X-100, 50 mM Tris-HCl (all from Sigma-Aldrich), and the selected formamide concentration [29]. The bacteria were fixed in 4% paraformaldehyde solution in PBS. Ten µL were added to each well on a microscope slide with six reaction wells (Marienfeld-Superior laboratory glassware) and air-dried at 37 °C. Samples were dehydrated by sequentially submersion in 50% ethanol, 80% ethanol and 96% ethanol, three minutes in each. Seven µL of hybridization buffer was added to each reaction well and 1 µL PNA-FISH probe was mixed with the hybridization buffer to a final probe concentration of 0.5 µM. The samples were incubated in a moist chamber at 55 °C for 90 min in the dark. Thereafter, the samples were washed in pre-heated wash solution (5 mM Tris base, 15 mM NaCl and 1% (vol/vol) Triton X-100 (Sigma-Aldrich) for 30 min at 55 °C, dipped once in cold milliQ water and air dried. Imaging was performed using confocal microscopy using the same settings for all images and the signal intensity of individual bacterial cells was measured using ImageJ [30]. The best condition for both sensitivity and specificity was achieved at 25% formamide.

### 2.7. Detection and Distribution of Achromobacter in Sputum Samples Obtained from CF Patients

Sputum samples were fixed with 4% paraformaldehyde solution, embedded in paraffin and sectioned to a thickness of 4–10 µm. Sections were mounted on microscope slides for confocal microscopy, dried for two hours at 60 °C and stored at −20 °C prior to analysis. Samples were investigated as previously described [31] with minor modifications. In brief, paraffin was removed by immersing the sample sequentially in xylene (2 × 5 min), 99% ethanol (2 × 3 min), 96% ethanol (2 × 3 min) and water (3 × 3 min). Samples were air-dried and stored at room temperature prior to analysis. Fourteen µL hybridization buffer (25% formamide) and two µL probe was added to the tissue samples to a final probe concentration of 0.5 µM. A cover slip was placed on top of each sample and samples were incubated in a moist chamber at 55 °C for 90 min in the dark. The cover slip was carefully removed and the samples were washed in pre-heated wash solution for 30 min at 55 °C. Samples were dipped once in cold water and air dried, followed by counter staining with Syto 41 (10 µM in water, Sigma). Imaging was performed by confocal microscopy in λ mode, using a reference spectrum obtained from pure cultures of *Achromobacter* hybridized with the *Achromobacter* specific PNA-FISH probe and stained with Syto 41.

### 2.8. Enzyme Treatment to Prevent Biofilm Formation

Overnight cultures were grown in BHI and adjusted to OD600 = 0.1 with fresh BHI, and 160 µL per well was added to 96 well micro titer plates One of the following enzymes was added before peg-lids were inserted: DNase I (Deoxyribonuclease I from bovine pancreas, 2297 Kunits/g), alginate lyase (alginate lyase from Sphingobacterium multivorum, 10.000 U/g) or subtilisin (Protease from Bacillus licheniformis, 9 U/µg). The two concentrations used were 50 µg/mL (low) and 200 µg/mL (high). After 24 h incubation at 37 °C, biofilm formation in the presence of each enzyme was quantified by crystal violet staining and compared to controls with no addition of enzyme. The experiments were carried out three times with eight replicates in each experiment.

### 2.9. Enzyme Treatment of Mature Biofilms

Biofilms were formed on peg-lids as described above, except in the absence of enzymes. After 24 h incubation, the peg-lid was transferred to a new plate containing sterile BHI media supplemented with DNaseI, alginate lyase or subtilisin. Each enzyme was administered in two concentrations; 50 µg/mL (low) and 200 µg/mL (high). The biofilms were treated for two hours at 37 °C before quantification by crystal violet staining. The experiments were carried out three times with eight replicates in each experiment.

### 2.10. Antimicrobial Susceptibility Testing

Antimicrobial susceptibility was tested for ceftazidime, colistin, piperacillin/tazobactam and tobramycin to evaluate the antibiotic tolerance of biofilms compared to planktonic cultures of the same isolate.

The minimal inhibitory concentration (MIC) was determined using Etest (Biomérieux) according to the manufacturer’s protocol. The minimal biocidal concentration (MBC) was determined by twofold serial dilutions ranging from 1024 µg/mL to 1 µg/mL. Overnight cultures in BHI were adjusted to OD600 = 0.2 with fresh media and 100 µL/well was added to 96 well plates (Nunc, Nunclon Delta Surface, Thermo Fischer Scientific, Waltham, MA, USA). Antibiotics were dissolved in sterile growth media to a concentration twice that of the desired concentration and 100 µL were added to each well already containing 100 µL bacterial culture, and mixed by pipetting. Antibiotic-free growth controls and bacteria-free blanks were included on each plate. The plate was incubated statically for 24 h at 37 °C and OD600 was measured. From each well with no visual growth, 100 µL was plated on 5% blood agar and incubated overnight at 37 °C. MBC was defined as the concentration of antibiotics that yielded a reduction in CFU count of ≥99.9%.

Biofilms were formed on peg-lids in BHI media and incubated for 24 h at 37 °C and transferred to a micro titer plate containing sterile BHI media supplemented with ceftazidime, colistin, piperacillin/tazobactam or tobramycin (Sigma-Aldrich) in twofold serial dilutions ranging from 8192 µg/mL to 128 µg/mL. The effect of enzyme treatment on MBEC was measured by addition of DNase I (200 µg/mL), alginate lyase (200 µg/mL) or subtilisin (200 µg/mL) to the micro titer plate supplemented with antimicrobials. After 24 h incubation at 37 °C, the peg-lid was gently rinsed in sterile media and transferred to a new plate containing fresh, sterile media and incubated for 24 h at 37 °C. Following incubation, the optical density OD600 was measured and from wells with no apparent growth, 100 µL was plated on 5% blood agar and incubated for 24 h at 37 °C. The wells with no growth were used to identify the minimal concentration of antibiotic needed to completely eradicate the biofilm. The experiments were carried out three times with three replicates in each experiment.

### 2.11. Statistical Analysis

Statistical analysis was performed using SigmaPlot (Systat Software, San Jose, CA, USA). Since data was not normally distributed, a Wilcoxon Mann-Whitney rank-sum test was applied to compare biofilm formation of isolates and to investigate the effect of enzymatic treatment. A *p*-value < 0.05 was considered statistically significant.

## 3. Results

### 3.1. Clinical Achromobacter Isolates form Biofilm in Vitro

**Figure 1:**
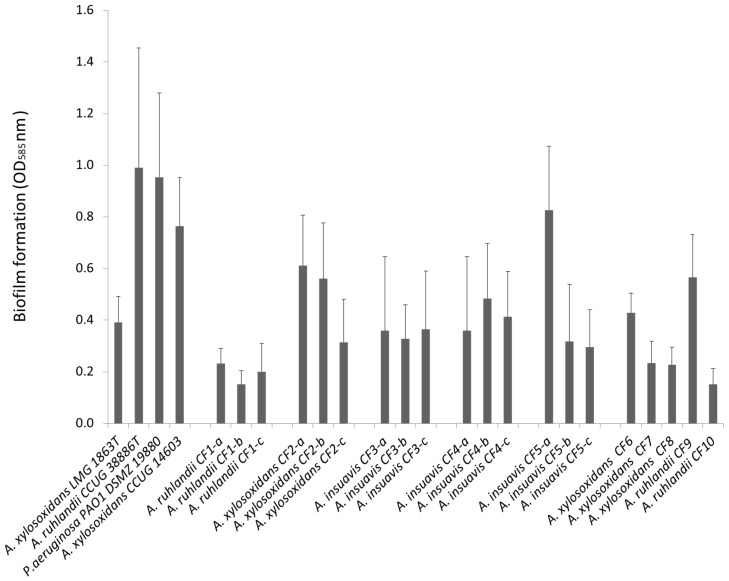
Mean production of biofilm ± SD in 23 *Achromobacter* isolates and *P. aeruginosa* DSMZ 19880 measured by crystal violet staining (strains are listed in Table 1).

The ability to form biofilm is believed to be of crucial importance for establishing chronic lung infections in cystic fibrosis patients. We therefore screened a selection of clinical and non-clinical *Achromobacter* isolates to evaluate their ability to form biofilm in vitro. We investigated 23 *Achromobacter* strains; three from culture collections and twenty clinical isolates from CF patients. A fast screening using a crystal violet micro titer peg-lid assay shows that all 23 isolates formed biofilm in BHI media (Figure 1). *A. ruhlandii* CCUG 38886^T^ (type strain), *A. xylosoxidans* CCUG 14603 (environmental isolate), and the clinical isolates *A. ruhlandii* CF9 and *A. insuavis* CF5-a displayed a biofilm forming capacity similar to PAO1, whereas all other isolates produced less biofilm. Our study included three species of *Achromobacter*, and the observed variation in biofilm formation could not be ascribed to a specific species. Three separate isolates were investigated from five patients, and production of attached biofilm was generally highest pronounced for first-time isolates (Figure 1), the reduction in biofilm formation for later isolates attained statistical significance for CF5 and CF2 (*p* > 0.001). Of the clinical isolates, *A. insuavis* CF5-a produced the largest amount of attached biofilm, more than five times that of *A. ruhlandii* CF1-b which produced the lowest amount of attached biofilm. *A. ruhlandii* CF9 and *A. ruhlandii* CF10 both belong to the same strain (DES), but are isolated from different patients. CF9 represents an early isolate, whereas *A. ruhlandii* CF10 represents a late isolate. More attached biofilm is produced by the early isolate, consistent with the findings from CF2 and CF5. *A. xylosoxidans* CF6 and CF8 represent early isolates, whereas CF7 represents a late isolate (approximate 6 years after first acquisition). Production of attached biofilm varied amongst clinical *Achromobacter* isolates and did not appear to be species specific, rather strain and even isolate specific.

The reduction in biofilm formation in isolates was not caused by reduced in vitro growth rates in later isolates of the same strain, though variation between strains was observed (Figure S1). Two clinical isolates from chronic infections were chosen for further investigation, namely *A. xylosoxidans* CF2-b characterized by high production of attached biofilm and *A. insuavis* CF4-b characterized by low level production of attached biofilm.

### 3.2. Biofilm Structure, Morphology and Extracellular Matrix Composition

**Figure 2:**
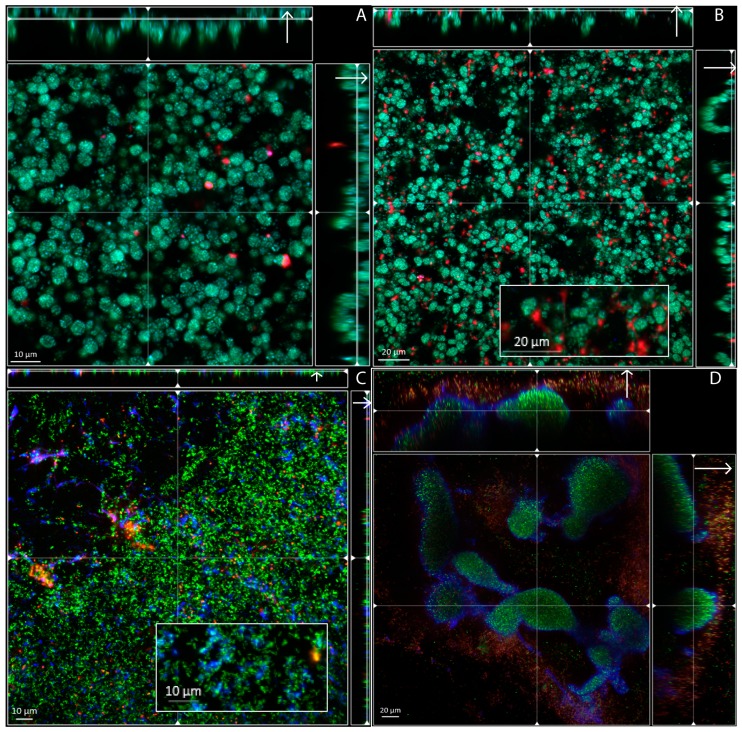
Biofilm structure and matrix composition of *A. xylosoxisans* LMG 1863^T^ and clinical isolates *A. xylosoxidans* CF2-b and *A. insuavis* CF4-b. Biofilms were grown for 48 h at continuous flow (**A**–**C**) and at static conditions (**D**) and visualized by confocal microscopy. Living cells were stained with Syto 9 (green), dead cells and extracellular DNA with propidium iodide (red) and polysaccharides were stained with fluorescent brightener (blue). *A. xylosoxidans* LMG 1863^T^ (**A**) and *A. xylosoxisans* CF2-b (**B**) produced surface attached biofilm, whereas *A. insuavis* CF4-b (**C**) failed to form biofilm under continuous flow, but formed suspended aggregates encased in polysaccharides (blue) when grown at static conditions (**D**).

*A. xylosoxidans* CF2-b, *A. insuavis* CF4-b and the type strain of *A. xylosoxidans* LMG 1863^T^, were grown under continuous flow (Figure 2). *A. xylosoxidans* LMG 1863^T^ and *A. xylosoxidans* CF2-b produced surface-attached biofilms of similar morphology (Figure 2A,B) containing aggregates of up to 5 µm in diameter within the biofilm measuring up to 20 µm in thickness. In the biofilm produced by *A. xylosoxidans* CF2-b, eDNA (red) appeared in clumps between bacterial aggregates throughout the biofilm (Figure 2B). This was not observed for *A. xylosoxidans* LMG 1863^T^, despite the otherwise phenotypically similar appearance of the biofilms produced by the two isolates. Staining of *A. xylosoxidans* LMG 1863^T^ and *A. xylosoxidans* CF2-b aggregates (Figure 2A,B) suggest co-localization of syto 9 (living cells) and calcofluor (polysaccharides). This was not observed for *A. insuavis* CF4-b (Figure 2C,D), suggesting strain-dependent differences in polysaccharide localisation. *A. insuavis* CF4-b did not form a surface attached biofilm under flow, and only a few layers of cells and clumps of polysaccharides (blue) were observed (Figure 2C). When grown under static conditions, however, *A. insuavis* CF4-b displayed a loosely attached or unattached biofilm phenotype of suspended aggregates of up to approximately 80 µm (Figure 2D). The aggregates were encased in an extracellular matrix of polysaccharides (blue) forming a peripheral shell around the aggregates. This phenomenon was not observed with the two *A. xylosoxidans* isolates (data not shown). Attachment to an abiotic surface is not necessarily required for *Achromobacter* biofilm formation, and unattached aggregates may possibly be an occurring mode of growth in vivo in sputum within the CF lung.

### 3.3. Visualization of Achromobacter in Sputum Samples from Cystic Fibrosis Patients, Exhibit Resemblance with Biofilm Aggregates

**Figure 3:**
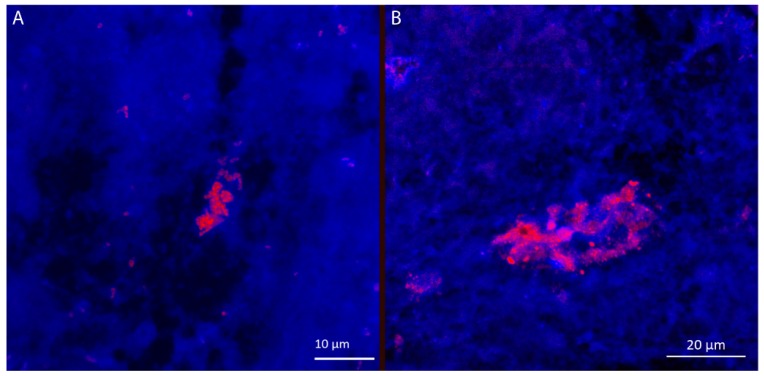
*Achromobacter* aggregates in sputum samples from CF patients CF2 and CF4 visualized by PNA-FISH *Achromobacter*-specific probe (red). The sample was counter-stained with Syto 41 (blue). The aggregates were 5–10 µm in diameter in patient CF2 (**A**), and up to 40 µm in patient CF4 (**B**). The distribution of aggregates was heterogeneous, and the images do not reflect the general abundance of *Achromobacter* in the sample.

*Achromobacter* has been shown to form biofilm in vitro [11,12], but the mode of growth in vivo has yet to be fully elucidated. To investigate the formation of in vivo biofilm, sputum samples from patient CF2 and CF4 were collected in early 2015. *Achromobacter*-specific PNA-FISH probes was used to visualize the spatial distribution of these bacteria within the sputum samples. The specificity of the probe was validated in vitro showing no unspecific binding to the selected control strains at the formamide concentration used. *Achromobacter* sp. were grown from both sputum samples and aggregates were visualized in the sputum by PNA-FISH (Figure 3). The aggregates were heterogeneously distributed in the samples and reached a diameter of 5–10 µm for patient CF2 and up to 40 µm for patient CF4. The aggregate morphology was consistent with our in vitro observations; however, *Achromobacter* aggregates visualized in sputum were generally smaller than observed in vitro.

### 3.4. Extracellular Matrix Components Are Important for Formation and Stability of Achromobacter Biofilm

**Figure 4:**
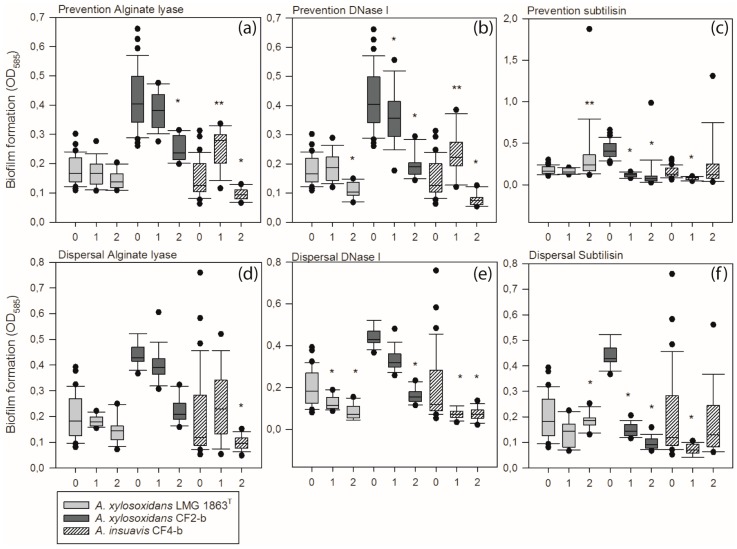
Enzymatic treatment both reduced and increased *Achromobacter* biofilm formation and caused biofilm dispersal. Enzymatic treatment during biofilm formation (**a**–**c**, prevention) and enzymatic treatment of 24 h old biofilms (**d**–**f**, dispersal) in *A. xylosoxidans* LMG 1863^T^, *A.* *xylosoxidans* CF2-b and *A. insuavis* CF4-b. Biofilms were subjected to treatment with alginate lyase (**a** and **d**), DNase (**b** and **e**) and subtilisin (**c** and **f**). 0: untreated, 1: low concentration of enzyme (50 µg/mL), 2: high concentration of enzyme (200 g/mL) * Statistically significant reduction compared to untreated. ** Statistically significant increase compared to untreated. A *p*-value < 0.05 was considered statistically significant.

The ability of bacteria to form biofilm depends on various factors, amongst which production of the extracellular matrix is of key importance for the biofilm structure and stability. To examine the importance of various biomolecules for development and maintenance of biofilm structure and stability in *Achromobacter*, we removed extracellular DNA (eDNA), protein or alginate by enzymatic treatment with DNase, subtilisin, or alginate lyase. The effect of EPS-degrading enzymes on formation of attached biofilm was investigated by adding the enzymes simultaneous with inoculating bacteria for biofilm growth (Figure 4a–c). The degradation of alginate by alginate lyase (200 µg/mL) reduced the amount of attached biofilm produced by *A. xylosoxidans* CF2-b (41%, *p* = 0.01) and *A. insuavis* CF4-b (34%, *p* < 0.001), whereas alginate lyase had no effect on the amount of attached biofilm in *A. xylosoxidans* LMG 1863^T^. Degradation of eDNA by DNase I (200 µg/mL) reduced the amount of attached biofilm produced by all three isolates (*A. xylosoxidans* LMG 1863^T^ with 38%, *p* < 0.00; CF2-b with 53%, *p* < 0.001 and CF4-b with 48%, *p* = 0.004) thus having the greatest overall effect on limiting the formation of attached biofilm. Surprisingly, addition of DNase I at 50 µg/mL significantly increased, rather than decreased, the amount of attached biofilm formed by *A. insuavis* CF4-b (38%, *p* = 0.001). The addition of 50 µg/mL subtilisin reduced the amount of biofilm formed by *A. xylosoxidans* CF2-b (72%, *p* < 0.001) and *A. insuavis* CF4-b (48%, *p* < 0.001), whereas 200 µg/mL subtilisin increased the amount of biofilm formed by *A. xylosoxidans* LMG 1863^T^ (67%, *p* = 0.0154).

To investigate the importance of various biomolecules for maintenance of matured biofilms, we investigated if 2 h enzyme treatment of 24 h old biofilms would lead to dispersal (Figure 4d–f). DNase I treatment resulted in a significant reduction in attached biofilm for all isolates (*A. xylosoxidans* LMG 1863^T^ with 57%, *p* = 0.0017; *A. xylosoxidans* CF2-b with 51%, *p* = 0.0026 and *A. insuavis* CF4-b with 63%, *p* = 0.0015) (Figure 4e). In contrast, treatment with subtilisin showed larger variability between strains, with a reduction of attached biofilm of up to 84% for *A. xylosoxidans* CF2-b (*p* < 0.001) and as low as 6% (*p* = 0.0084) for In *A. xylosoxidans* LMG 1863^T^ (Figure 4f). The effect of alginate lyase was restricted to *A. insuavis* CF4-b, where a reduction in attached biofilm of 52% (*p* = 0.0044) was observed (Figure 4d).

### 3.5. Increased Antimicrobial Tolerance of Bacteria Embedded in Biofilm

Biofilm formation greatly increases the amount of antibiotics needed to eradicate bacteria. Antimicrobial susceptibility testing was performed on isolates *A. xylosoxidans* LMG 1863^T^, *A. xylosoxidans* CF2-b and *A. insuavis* CF4-b in order to test how the biofilm mode of growth affected the efficacy of antimicrobials. Tolerance to the tested antimicrobials increased dramatically for all isolates when grown as biofilm (MBEC) when compared to planktonic cultures (MBC) (Table 2). Generally, MBEC was between eight to 1000 fold higher than MBC. Colistin had the greatest effect on biofilm eradication, with an MBEC of 128 µg/mL in all isolates and an increase of only eight to sixteen times MBC. The most pronounced increase in MBEC was found for piperacillin/tazobactam, greatest in *A. insuavis* CF4-b, with an MBEC of 2048 µg/mL and an increase of 1000 times MBC. We found no difference in MBEC between the two clinical isolates for colistin, piperacillin/tazobactam and tobramycin, but the MBEC value for ceftazidime was four times higher in *A. xylosoxidans* CF2-b compared to *A. insuavis* CF4-b. Antimicrobial tolerance increased in all isolates when was grown as biofilms, and the amount of antimicrobials needed to fully eradicate the biofilms was up to 1000 fold higher that for planktonic bacteria. Increased antimicrobial tolerance is likely to be the cause of many unsuccessful eradication attempts in chronically infected cystic fibrosis patients.

Because enzyme treatment could partially disperse biofilms, we investigated if treatment with subtilisin or DNase simultaneously with antibiotics would lead to a higher susceptibility to either colistin or piperacillin. While the MBEC for piperacillin remained unchanged for 2 of the 3 isolates, enzyme treatment did affect the MBEC for colistin in most cases (Table 2). Of the two enzymes tested, subtilisin appeared to be most effective, as subtilisin treatment reduced the MBEC for colistin for all 3 strains by 50%–75%, whereas DNase treatment led to a 50% reduction in the MBEC for 2 of the 3 strains. Enzymatic treatment did not reduce the MBEC of ceftazidime or tobramycin, nor did enzymatic treatment with alginate lyase reduce the MBEC for any of the tested antibiotics. Treatment with DNase or subtilisin partially dispersed the biofilm and reduced the tolerance to colistin or piperacillin, paving the way for further research into using dispersal mechanisms to improve treatment strategies.

## 4. Discussion

Biofilm formation is an important aspect of establishing and maintaining chronic infections within the lungs of cystic fibrosis patients. *Achromobacter xylosoxidans* has the capacity to form biofilm in vitro, yet little is known about its ability to form biofilm in vivo or of the structure, composition and spatial distribution of *Achromobacter* biofilms within the CF lung. In the present study, we quantified the amount of attached biofilm formed by clinical isolates of *Achromobacter* from CF patients and characterized important aspects of biofilm formation in selected isolates.

The quantification of biofilm formation in different *Achromobacter* isolates revealed no inter-species variation but rather a development over time after establishment of *Achromobacter* in the lung of a given CF patient. Generally, isolates obtained after 1–4 years of chronic infection have reduced capability to form attached biofilm in comparison with the initial isolate obtained from the same patient. The reduction in biofilm formation was not caused by reduced in vitro growth rates in later isolates, as has been seen in *P. aeruginosa*, where reduced biofilm formation was reflected in lower growth rates [32]. Thus, the reduced formation of attached biofilm may result from within-host adaptation to the CF lung [33]. A recent study [34] showed induction of aggregate formation and increased hydrophobicity in *P. aeruginosa* in the presence of anti-*Pseudomonas* antibodies, thereby increasing phagocytic clearance. Thus, it is possible that isolates that produce less attached biofilm when obtained after years of chronic infection may have an altered morphotype where aggregate formation is induced as a consequence of adaptive host mechanisms.

Biofilms are classically defined as structured bacterial communities attached to a surface and encased in a self-produced matrix [13]. However, surface attachment as a crucial part of biofilm formation has since been challenged, and it was later proposed to include non-attached aggregates in the biofilm definition [14]. In this study, we visualized in vitro biofilm growth, revealing distinctly different biofilm morphotypes between selected *Achromobacter* isolates. *A. xylosoxidans* LMG 1863^T^ and *A. xylosoxidans* CF2-b both produced surface attached biofilms comprised of spherical aggregates of approximately 5–10 µm in diameter (Figure 2a,b). A mesh-like structure of extracellular DNA similar to previous findings in *P. aeruginosa* [27] was seen with *A. xylosoxidans* CF2-b when grown under continuous flow, suggesting that eDNA may play a role in biofilm attachment in this isolate. In contrast, *A. insuavis* CF4-b formed large non-attached or loosely attached biofilm-like aggregates when grown under static conditions (Figure 2d), similar to previous reports of *P. aeruginosa*, *Staphylococcus aureus* and some strains of *A. xylosoxidans* biofilms [11,14,35,36]. The biofilm aggregates appeared to be held together by polysaccharides forming a peripheral shell around the bacterial cells. *A. insuavis* CF4-b did not form attached biofilm under flow (Figure 2c), as un-attached bacteria were quickly removed from the flow cells. Our results highlight the importance of critically evaluating the in vitro model used for studying biofilm production by bacteria cultured from chronic infections, where the absence of attachment to an abiotic surface may lead to falsely concluding that a clinical isolate is incapable of forming biofilm.

In contrast to in vitro biofilm formation under high nutrient and oxygen availability, biofilms within the CF airways form in dense mucus which may become oxygen depleted. In addition to oxygen and nutrient availability, interactions with the host immune system, competition from other bacterial species and antibiotic treatment may influence in vivo biofilm formation [37,38]. To investigate if our in vitro observations represented the morphology of in vivo biofilms, we visualized *Achromobacter* sp. in mucus collected from the patients from whom the isolates originated. Biofilm aggregates were heterogeneously scattered throughout the sputum samples, and were much smaller than the aggregates formed in vitro. The aggregates were 5–10 µm in diameter in sputum collected patient CF2 (Figure 4a), whereas most aggregates in sputum from patient CF4 were up to approximately 40 µm in diameter (Figure 4b). The aggregate morphology is consistent with our in vitro observations and with previous findings showing biofilm-like aggregate structures in sputum from CF patients using Gram-stain [12]. In a review focusing on in vivo biofilms [38], two main differences in biofilm morphology between in vitro and in vivo biofilms in chronic infections was pointed out. Firstly, the mushroom structure typical for *P. aeruginosa* when grown in flow cells is not observed in vivo and secondly, the size of biofilm aggregates is generally smaller in vivo. In accordance with this, *Achromobacter* aggregates visualized in sputum were generally smaller than observed in vitro.

The biofilm phenotype is characterized by sessile bacteria with lowered metabolic activity encased in an extracellular matrix [13]. These characteristics render biofilms highly tolerant towards antimicrobials and shield the bacteria from the host immune system. Expectedly, we found an increase in antimicrobial tolerance when *Achromobacter* was grown as biofilms, and the amount of antibiotics needed to fully eradicate the biofilm was up to 1000 times higher than for planktonic cultures (Table 2). Colistin is commonly used for treatment of cystic fibrosis. It targets the cells in the inner, dormant part of the biofilm [39]. It can be administered as aerosolized antimicrobial inhalation therapy, thereby achieving high local concentrations in sputum [40]. Nebulized colistin is used in Danish CF centers and has been shown to prevent or postpone the development of chronic infection with *Achromobacter* [41] and *P. aeruginosa* [42]. A recent study tested the effect of high-dose antimicrobials on biofilm growth on three *Achromobacter* species from cystic fibrosis patients. The study showed that levofloxacin and tobramycin had the greatest overall efficacy [19]. In this study, we found that colistin had the lowest MBEC (128 µg/mL) of the tested antimicrobials (Table 2). This may be due to varying antimicrobial resistance caused by differences in treatment schemes at the local cystic fibrosis centers, as well as variation in in vitro growth conditions.

Despite aggressive antimicrobial therapy, attempts to eradicate chronic infections in cystic fibrosis patients are often unsuccessful. Attention has therefore turned to disrupting the biofilm matrix with the intent to increase the penetration of antimicrobials into the biofilm and to disperse bacteria from within the biofilm, rendering them susceptible to treatment. Since 1994, inhalation therapy using human recombinant DNase has been used in treatment of cystic fibrosis patients, reducing the viscosity of the sputum [43,44] and disrupting eDNA accumulating in the lungs [45]. Today, daily DNase inhalation with Pulmozyme™ (dorenase alfa) is standard treatment in Danish CF centers, where it has been proved beneficial to patients both with and without chronic pulmonary infection, improving lung function and reducing the demand for antimicrobials [46,47].

We show here that eDNA is also important for in vitro *Achromobacter* biofilm formation. Something which is also true for numerous other Gram-positive and Gram-negative bacteria [48,49]. The ability of DNase to disperse biofilm usually disappears within the first 12–24, and our study presents the first example of DNase-mediated biofilm dispersal in mature (24 h old) *Achromobacter* biofilms (Figure 4). Until the biofilm reaches a certain age, DNA has been shown to disperse biofilm in other species [50,51]. It is not known whether this age-dependency applies to *Achromobacter* biofilms. DNase I and subtilisin were able to disperse biofilms of all three isolates, whereas alginate lyase only caused dispersal in *A. insuavis* CF4-b. The presence of enzymes from inoculation of the strain during 24 h of biofilm development did in most cases reduce the generation of biofilm but could not completely inhibit the biofilm formation (Figure 4a–c). Likewise, challenging the matrix of a mature (24 h) biofilm with enzymes reduced biofilm biomass but did not eradicate the biofilm (Figure 4d–f). However, our in vitro results suggests that combining antibiotic and enzymatic treatment has the potential to help combatting *Achromobacter* biofilms in vivo by reducing the amount of antibiotics needed to eradicate the biofilm.

Polysaccharides, such as alginate, are also an important component of the biofilm matrix, and enzymatic treatment with alginate lyase has thus been shown to cause dispersal of *Pseudomonas* *aeruginosa* [52], whereas enzymatic treatment with DNase I and proteinase impair biofilm formation and cause dispersal in *Listeria monocytogenes* [53]. Even though an effect of alginate lyase was shown on biofilm formation (*A. xylosoxidans* CF2-b and *A. insuavis* CF4-b) and dispersal (*A. insuavis* CF4-b only) (Figure 4), no reduction in MBEC could be measured (Table 2). The alginate biosynthesis protein AlgJ is present in the genome of *A. xylosoxidans* CF2b, however, as it is still not fully elucidated whether all *Achromobacter* sp. produce alginate as part of the extracellular matrix, or whether a different exopolysaccharide may be partially degraded by alginate lyase, other polysaccharide lyases might be more effective in combination with antimicrobial treatment.

The fact that treatment with DNase I or subtilisin reduced the MBEC of colistin and that enzyme treatment with DNase I, alginate lyase or subtilisin all showed a reduction in biofilm biomass in *Achromobacter* biofilms paves the way for further research into using dispersal mechanisms, possibly targeting several matrix components, to improve antimicrobial treatment strategies in chronic *Achromobacter* infections in cystic fibrosis patients. A better understanding of the capacity of *Achromobacter* to form biofilm may help improve diagnosis and treatment strategies of *Achromobacter* in cystic fibrosis patients. A previous study [21] investigated SNP’s in whole-genome sequences of CF1–CF5. It was apparent from this study that isolates CF2 and CF4 had not accumulated large amounts of mutations (CF2 = 29 and CF4 = 17) when compared to the number of SNP’s found in CF5 (CF5 = 224) even though we found significant variation in biofilm formation at the isolate level in CF2 as well as CF5. We therefore hypothesize that changes in isolates from primary and chronic infections may be caused by altered expression of genes related to biofilm formation rather than mutations at the DNA level. Targets for parallel selection in *P. aeruginosa* biofilms include genes involved in type IV pilus motility, flagellar motility, biofilm formation, metabolism and regulation [54] Likely these genes will also be of interest when studying evolution of *Achromobacter* biofilms. Further studies elucidating the genetic mechanisms involved in *Achromobacter* biofilm formation may provide valuable insight into understanding the underlying mechanisms involved in biofilm formation and persistence in chronic infections.

## Figures and Tables

**Table 1:** Bacterial strains used in this study.

Species	Isolate	Strain Identification	Origin	Source
*A. xylosoxidans*	Type strain	LMG 1863^T^	Osaka, Japan	Ear
*A. ruhlandii*	Type strain	CCUG 38886^T^		Soil
*P. aeruginosa*	PAO1	DSMZ 19880		
*A. xylosoxidans*	Environmental isolate	CCUG 14603	Lyon, France	Water
*A. ruhlandii*	CF1-a	J9557-07	Aarhus, Denmark	CF clinical isolate
*A. ruhlandii*	CF1-b	J4616-09	Aarhus, Denmark	CF clinical isolate
*A. ruhlandii*	CF1-c	J4951-12	Aarhus, Denmark	CF clinical isolate
*A. xylosoxidans*	CF2-a	J19840-07	Aarhus, Denmark	CF clinical isolate
*A. xylosoxidans*	CF2-b	J19373-08	Aarhus, Denmark	CF clinical isolate
*A. xylosoxidans*	CF2-c	J8663-11	Aarhus, Denmark	CF clinical isolate
*A. insuavis*	CF3-a	J10719-08	Aarhus, Denmark	CF clinical isolate
*A. insuavis*	CF3-b	J13140-09	Aarhus, Denmark	CF clinical isolate
*A. insuavis*	CF3-c	J13665-10	Aarhus, Denmark	CF clinical isolate
*A. insuavis*	CF4-a	J14174-08	Aarhus, Denmark	CF clinical isolate
*A. insuavis*	CF4-b	J10317-09	Aarhus, Denmark	CF clinical isolate
*A. insuavis*	CF4-c	J1036-12	Aarhus, Denmark	CF clinical isolate
*A. insuavis*	CF5-a	J15059-09	Aarhus, Denmark	CF clinical isolate
*A. insuavis*	CF5-b	J23156-10	Aarhus, Denmark	CF clinical isolate
*A. insuavis*	CF5-c	J5762-12	Aarhus, Denmark	CF clinical isolate
*A. xylosoxidans*	CF6	J20454-08	Aarhus, Denmark	CF clinical isolate
*A. xylosoxidans*	CF7	J15887-08	Aarhus, Denmark	CF clinical isolate
*A. xylosoxidans*	CF8	J15976-09	Aarhus, Denmark	CF clinical isolate
*A. ruhlandii*	CF9	J18469-02	Aarhus, Denmark	CF clinical isolate
*A. ruhlandii*	CF10	J10633-12	Aarhus, Denmark	CF clinical isolate

**Table 2:** Antimicrobial susceptibility of *A. xylosoxidans* LMG 1863^T^, *A. xylosoxidans* CF2-b and *A. insuavis* CF4-b.

	*A. xylosoxidans* LMG 1863^T^			*A. xylosoxidans* CF2-b			*A. insuavis* CF4-b		
Antibiotic (µg/mL)	MIC	MBC	MBEC	MIC	MBC	MBEC	MIC	MBC	MBEC
Piperacillin/tazobactam	1	8	1024	1	8	2048	1	2	2048
+ DNase I (200 µg/mL)	-	-	1024	-	-	2048	-	-	512
+ Subtilisin (200 µg/mL)	-	-	1024	-	-	2048	-	-	512
+ Alginate lyase (200 µg/mL)	-	-	1024	-	-	2048	-	-	2048
Ceftazidime	8	16	2048	8	8	2048	4	8	512
+ DNase I (200 µg/mL)	-	-	2048	-	-	2048	-	-	512
+ Subtilisin (200 µg/mL)	-	-	2048	-	-	2048	-	-	512
+ Alginate lyase (200 µg/mL)	-	-	2048	-	-	2048	-	-	512
Tobramycin	>256	1024	1024	48	128	1024	24	64	1024
+ DNase I (200 µg/mL)	-	-	1024	-	-	1024	-	-	1024
+ Subtilisin (200 µg/mL)	-	-	1024	-	-	1024	-	-	1024
+ Alginate lyase (200 µg/mL)	-	-	1024	-	-	1024	-	-	1024
Colistin	4	8	128	4	8	128	4	16	128
+ DNase I (200 µg/mL)	-	-	128	-	-	32	-	-	64
+ Subtilisin (200 µg/mL)	-	-	64	-	-	32	-	-	64
+ Alginate lyase (200 µg/mL)	-	-	128	-	-	128	-	-	128

-, not tested.

## Supplementary Materials

The following are available online at www.mdpi.com/2076-2607/4/3/33/s1, Figure S1: Growth curves of *A. xylosoxidans* LMG 1863^T^, *A. ruhlandii* CF1b, *A. xylosoxidans* CF2b, *A. xylosoxidans* CF2c, *A. insuavis* CF3b, *A. insuavis* CF4b, *A. insuavis* CF5a and *A insuavis* CF5b.
