# Supplementary material for: Achromobacter Species Isolated from Cystic Fibrosis Patients Reveal Distinctly Different Biofilm Morphotypes
Source: Microorganisms. 2016 Sep 14;4(3):33. doi: 10.3390/microorganisms4030033 (PMC5039593; doi:10.3390/microorganisms4030033)
Supplement: Supplementary file 1 [file microorganisms-04-00033-s001.pdf]

Supplementary Materials: *Achromobacter* Species Isolated from Cystic Fibrosis Patients Reveal Distinctly Different Biofilm Morphotypes

Signe M. Nielsen, Niels Nørskov-Lauritsen Thomas Bjarnsholt and Rikke L. Meyer

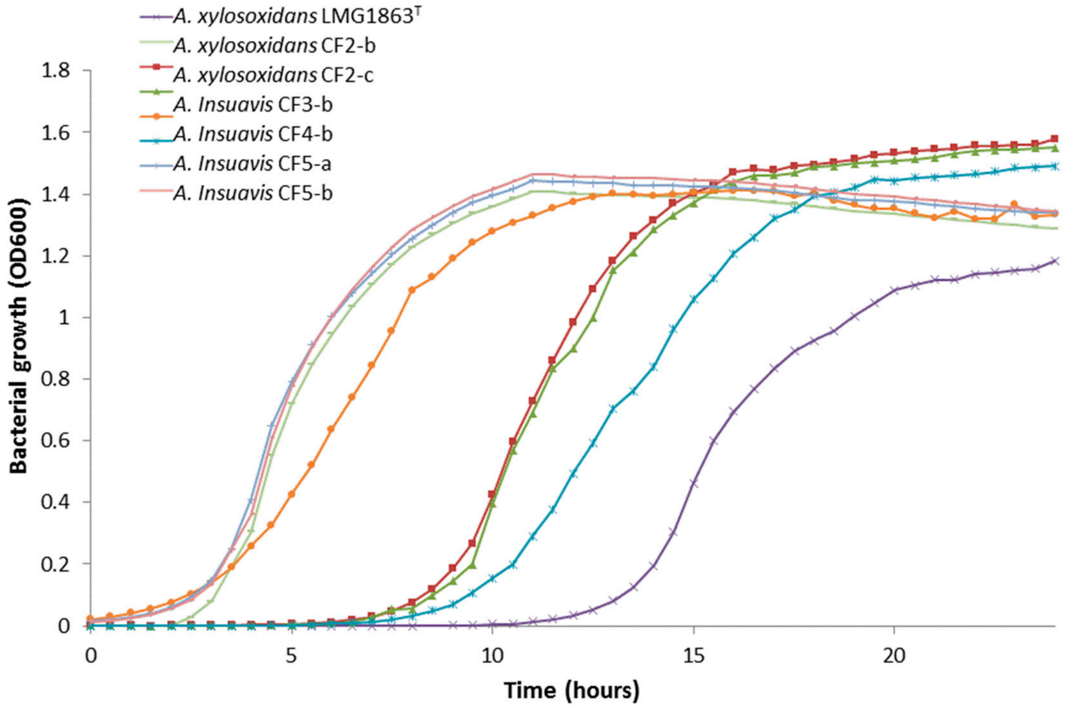

Figure S1. Growth curves of *A. xylosoxidans* LMG 1863^{T}, *A. ruhlandii* CF1b, *A. xylosoxidans* CF2b, *A. xylosoxidans* CF2c, *A. insuavis* CF3b, *A. insuavis* CF4b, *A. insuavis* CF5a and *A insuavis* CF5b.

© 2016 by the authors; licensee MDPI, Basel, Switzerland. This article is an open access article distributed under the terms and conditions of the Creative Commons by Attribution (CC-BY) license (http://creativecommons.org/licenses/by/4.0/).
